# Spatial distribution and cellular composition of adult brain proliferative zones in the teleost, *Gymnotus omarorum*

**DOI:** 10.3389/fnana.2014.00088

**Published:** 2014-09-08

**Authors:** Valentina Olivera-Pasilio, Daniel A. Peterson, María E. Castelló

**Affiliations:** ^1^Neurociencias Integrativas y Computacionales, Instituto de Investigaciones Biológicas Clemente EstableMontevideo, Uruguay; ^2^Neuroscience, Center for Stem Cell and Regenerative Medicine, Rosalind Franklin University of Medicine and ScienceNorth Chicago, IL, USA

**Keywords:** thymidine analogs, adult cell proliferation, weakly electric fish, electrosensory, teleosts

## Abstract

Proliferation of stem/progenitor cells during development provides for the generation of mature cell types in the CNS. While adult brain proliferation is highly restricted in the mammals, it is widespread in teleosts. The extent of adult neural proliferation in the weakly electric fish, *Gymnotus omarorum* has not yet been described. To address this, we used double thymidine analog pulse-chase labeling of proliferating cells to identify brain proliferation zones, characterize their cellular composition, and analyze the fate of newborn cells in adult *G. omarorum*. Short thymidine analog chase periods revealed the ubiquitous distribution of adult brain proliferation, similar to other teleosts, particularly *Apteronotus leptorhynchus*. Proliferating cells were abundant at the ventricular-subventricular lining of the ventricular-cisternal system, adjacent to the telencephalic subpallium, the diencephalic preoptic region and hypothalamus, and the mesencephalic tectum opticum and torus semicircularis. Extraventricular proliferation zones, located distant from the ventricular-cisternal system surface, were found in all divisions of the rombencephalic cerebellum. We also report a new adult proliferation zone at the caudal-lateral border of the electrosensory lateral line lobe. All proliferation zones showed a heterogeneous cellular composition. The use of short (24 h) and long (30 day) chase periods revealed abundant fast cycling cells (potentially intermediate amplifiers), sparse slow cycling (potentially stem) cells, cells that appear to have entered a quiescent state, and cells that might correspond to migrating newborn neural cells. Their abundance and migration distance differed among proliferation zones: greater numbers and longer range and/or pace of migrating cells were associated with subpallial and cerebellar proliferation zones.

## Introduction

In contrast to mammals, the adult central nervous system of teleosts exhibits a widespread distribution of proliferation zones with high neurogenic potential (Lindsey and Tropepe, 2006; Chapouton et al., 2007; Sullivan et al., 2007; Kaslin et al., 2008; Grandel and Brand, 2013). Neurogenesis is a process which involves cell proliferation, migration to target brain areas and differentiation into new neurons (Nowakowski and Hayes, 2008). Whereas in adult mammals *in vivo* neurogenesis occurs in two brain regions, the subventricular zone of the lateral ventricle (SVZ) and the subgranular zone of the hippocampal dentate gyrus (Altman and Das, 1965; Altman, 1969, 2011), in adult teleosts it occurs not only in their homologous structures but also in several other regions of all brain divisions (Lindsey and Tropepe, 2006; Zupanc, 2006, 2008; Chapouton et al., 2007; Kaslin et al., 2008; Grandel and Brand, 2013). Among teleosts, adult cell proliferation and neurogenesis have been thoroughly characterized in wave type weakly electric gymnotids, particularly in *Apteronotus leptorhynchus* [*A. leptorhynchus*: (Zupanc and Horschke, 1995; Zupanc et al., 1996; Zupanc, 2001; Hinsch and Zupanc, 2006); *Eigenmannia* sp: (Zupanc and Zupanc, 1992); and *Brachyhypopomus gauderio*: (Dunlap et al., 2011)]. The spatial distribution of brain proliferation zones in adult wave type weakly electric gymnotids roughly resembles that of other teleosts [*Astatotilapia burtoni* (Maruska et al., 2012); *Austrolebias* (Fernández et al., 2011); *Carassius auratus* (Raymond and Easter, 1983; Delgado and Schmachtenberg, 2011); *Danio rerio* (Maeyama and Nakayasu, 2000; Zupanc et al., 2005; Adolf et al., 2006; Grandel et al., 2006; Ampatzis and Dermon, 2007; Kaslin et al., 2009; Ito et al., 2010; März et al., 2010; Zupanc, 2011); *Gasterosteus aculeatus* (Ekström et al., 2001); *Nothobranchius furzeri* (Terzibasi et al., 2012); *Odontesthes bonariensis* (Strobl-Mazzulla et al., 2010); *Oreochromis mossambicus* (Teles et al., 2012); *Oryzias latipes* (Nguyen et al., 1999; Candal et al., 2005a; Alunni et al., 2010; Kuroyanagi et al., 2010; Isoe et al., 2012) and *Salmo trutta fario* (Candal et al., 2005b)], despite the phylogenetic distance to most of those species. However, differences have been observed that were attributed to the functional specialization of weakly electric fish (Zupanc and Horschke, 1995; Grandel et al., 2006; Grandel and Brand, 2013).

The brain of the pulse type weakly electric gymnotid *Gymnotus omarorum* also shows a widespread distribution of proliferation zones in late larvae (Iribarne and Castelló, 2014) and juveniles (Castelló and Iribarne, 2010) as evidenced by short chase of 5′ bromodeoxyuridine (BrdU), but the distribution of brain proliferation zones remains unexplored in adults. In late larvae and juveniles, brain regions involved in electrosensory processing show particular proliferation zones, either adjacent to the ventricular-cisternal system, for example the torus semicircularis (TS) and the tectum opticum (TeO), or distant from it [the electrosensory lateral line lobe (ELL) and the cerebellum (Cb)]. Mesencephalic and rombencephalic structures differ in their relative growth along postnatal development (Iribarne and Castelló, 2014), suggesting differences in the cellular composition, cell population dynamics, and/or neurogenic potential of their proliferation zones as occurs in mammals (Hermann et al., 2009).

Our objective was to characterize adult brain proliferation zones in *G. omarorum*. Here we show the qualitative distribution of adult *G. omarorum*'s brain proliferation zones and provide evidence for a new adult brain proliferation zone at the lateral-caudal border of the ELL. We analyzed the cellular composition of brain proliferation zones by means of a double thymidine analog labeling technique (Vega and Peterson, 2005).

All brain proliferation zones showed a heterogeneous cellular composition. Within the boundaries of all brain proliferation zones, we found abundant fast cycling proliferating cells and sparse slow cycling proliferating cells that probably correspond to amplifying progenitor and stem cells, respectively. Beyond the borders of brain proliferation zones, we also found long term weak thymidine analog label retaining cells that likely correspond to migrating newborn neural cells. Our results provide further evidences of conserved features of adult teleosts brain proliferation and form the basis for further analysis of survival and differentiation of newly-generated cells, as well as for the characterization of *G. omarorum* brain proliferation zones milieu throughout postnatal development. These findings contribute to understand the features that preserve adult neurogenic potential and subserve the continuous growth and high regenerative potential of teleosts brain. By providing this cytoarchitectural framework, we hope to facilitate future studies to elucidate the factors that modulate adult neurogenesis in amniotes.

## Materials and methods

### Animals

Seventeen adult specimens of *G. omarorum* (Richer-de-Forges et al., 2009) consisting of 11 females and 6 males (weight: 18.51 ± 10.2; length: 17.66 ± 4.03 cm) were collected from Laguna del Cisne, Uruguay (latitude 35°50′ S, longitude 55°08′ W). Animals were kept in individual tanks on a 12 h:12 h light:dark cycle and daily fed with *Tubifex tubifex*. Water conductivity was adjusted to 200 μS and temperature maintained at 24°C.

For intraperitoneal administration of drugs, fish were first anesthetized by immersion in ethyl 3-aminobenzoate methanesulfonate salt (MS-222, 120 mg/L Sigma, #A5040). For transcardial perfusion of fixative, fish were deeply anesthetized by immersion in MS-222 (500 mg/L) followed by gill perfusion of the same anesthetic solution.

All procedures were performed in accordance to the guidelines of CHEA (Comisión Honoraria de Experimentación Animal, ordinance number: 4332-99, Universidad de la República). Experiments were approved by the Animal Ethics Committee of the Instituto de Investigaciones Biológicas Clemente Estable (protocol number 010/09/2011).

### Thymidine analogs preparation and administration

The use of thymidine analogs has long been the most common tool for the study of cell proliferation, particularly within the nervous system (Gratzner, 1982; Taupin, 2007; Altman, 2011). More recently, the administration of two thymidine analogs discriminated by way of specific primary antibodies, along with experimental modification of the temporal windows between analogs administration (Vega and Peterson, 2005; Llorens-Martín and Trejo, 2011), has advanced the discrimination of proliferating cell subpopulations in the brains of amniotes (Vega and Peterson, 2005; Llorens-Martín et al., 2010), anamniotes (Grandel et al., 2006; Alunni et al., 2010) and invertebrates (Sullivan et al., 2007). To characterize the distribution and cellular composition of brain proliferation zones in adult *G. omaroum*, two thymidine analogs, 5′ chlorodeoxiuridine (CldU; Sigma, #C-6891) and 5′ iododeoxiuridine (IdU; MP Biomedicals, #100357) were administered according to two protocols (Vega and Peterson, 2005; Alunni et al., 2010). Equimolar solutions of CldU (2.3 mg/ml) and IdU (1.7 mg/ml) dissolved in 0.7% NaCl and 0.7% NaCl containing 0.04N NaOH, respectively, were administered i.p. at 25 μl/g. Each thymidine analog was given in a single injection. Thymidine analogs were sequentially administered with short (24 h, Protocol 1) or long (30 day, Protocol 2) chase between them. Animals were fixed after survival of 4 h (Protocol 1) or 24 h (Protocol 2) following the last thymidine analog administration.

### Fixation and immunohistochemistry

Animals were fixed by transcardial perfusion of 10% paraformaldehyde (PAF) dissolved in phosphate buffer 0.1 M, pH 7.4 (PB) preceded by saline perfusion to wash blood out from the circulatory system. Brains were dissected out and postfixed in the same fixative for 24 h at 4°C. After embedding in a gelatin/albumin mixture denatured with glutaraldehyde, frontal serial sections (60 μm) were obtained with a vibratome (Leica VT1000S, Wetzlar, Germany).

In order to expose the proliferation markers, tissue sections were pretreated to break double-stranded DNA into single strands, by incubation in 2 N HCl in PB containing 0.3% Triton X-100 (PB-T) for 50 min. After rinsing in PB (3 × 10 min), sections were incubated overnight at 40°C in both rat anti BrdU-CldU (AbD Serotec Cat# OBT0030, RRID:AB_609568; clone BU1/75; ICR1) and mouse anti BrdU-IdU (BD Biosciences Cat# 347580, RRID:AB_400326, Clone B44) at a dilution of 1:500 (in PB-T). Subsequently, sections were rinsed in PB (3 × 10 min) and incubated in donkey anti rat biotinylated secondary antibody at 1:500 (Jackson Immuno Research, # 712-065-153) in PBS-T for 1 h. After that, sections were rinsed 3 times in PB and incubated in a mixture of streptavidin Cy3 at 1:500 (Jackson, Cat#016-160-084) and donkey anti mouse Alexa 488 at 1:500 [Molecular Probes (Invitrogen) Cat# A21202, RRID:AB_141607] in PB-T for 90 min. After rinsing in PB (3 × 10 min), sections were mounted in PVA-DABCO coverslipping solution for immunofluorescence. Negative controls included omission of thymidine analog administration or omission of primary antibody incubation; both controls resulted in no detectable staining (data not shown).

### Antibody characterization

The mouse monoclonal anti-BrdU antibody, Clone B44, was first obtained by immunization of mice with IdU (Gratzner, 1982). The author proved that this antibody binds with high specificity with BrdU and IdU, does not cross-react with thymidine, and is useful to detect BrdU incorporated into the DNA of individual cells due to DNA replication. The rat anti-BrdU antibody, Clone BU1/75 (ICR1) binds with BrdU and CldU but not with IdU (Bakker et al., 1991; Aten et al., 1992). More recently, Vega and Peterson (2005) found that the mouse anti-BrdU antibody, but not the rat anti-BrdU monoclonal antibody detects IdU in tissue samples from animals exposed to this thymidine analog. On the other hand, the rat anti-BrdU antibody, but not the mouse anti-BrdU monoclonal antibody, detects CldU in tissue samples from animals exposed to this thymidine analog (Vega and Peterson, 2005). Cross reactivity of primary antibodies with the thymidine analogs was tested by incubation of sections of CldU only and IdU only treated animals with anti-IdU or anti-CldU antibodies, respectively (followed by incubation in the corresponding secondary antibodies; Supplementary Figure 1).

### Image acquisition and processing

Sections were imaged on a confocal system (Olympus BX61 microscope equipped with a FV300 confocal module and four lines of excitation: 405, 488, 543, and 633 nm). Acquisition settings were adjusted in order to ensure the use of the whole dynamic range of detection. Images correspond to 30 confocal planes that were sequentially scanned every 1 μm, and projected to one plane. If needed, postacquisition modifications were limited to small changes in the distribution histogram to achieve full use of the dynamic range. Co-expression of markers was confirmed by orthogonal projections in the *X-Z* and *Y-Z* planes.

### Mapping qualitative distribution

For mapping the spatial distribution of brain proliferation zones we obtained low power images of coronal sections (every 700 μm) stained with the Nissl technique. Briefly, brain serial sections were mounted on gelatine-coated slides, dried at room temperature and stained with 1% methylene blue. After dehydration in an ethanol series, the preparations were cleared in xylene and permanently mounted with Canada balsam. A dissecting microscope (AmScope, Irvine, CA, USA) equipped with a digital camera (DCM130, LabOptix, Sale, UK) was used to acquire 2-D images of all selected sections. A schematic diagram of each section was drawn using Inkscape, and the location of actively cells cycling (4 and 24 h after thymidine analog administration) and long term weak thymidine analog label retaining cells were represented by red and blue dots, respectively. The nomenclature and abbreviations used in this article to describe the location of brain proliferation zones in *G. omarorum* correspond to those used by Maler et al. (1991), Zupanc et al. (1996) and Meek and Nieuwenhuys (1998).

## Results

### General characteristics of nuclear labeling and methodological considerations

**Figure 1:**
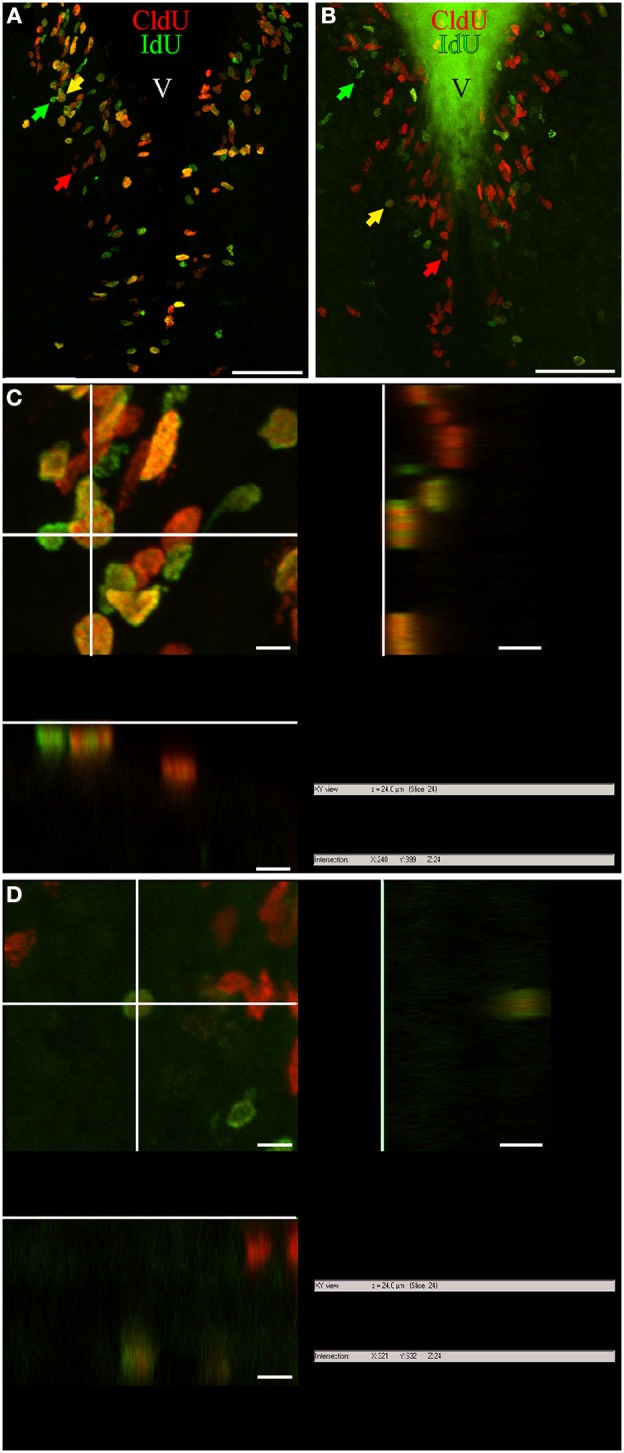
**Cellular composition of adult *G. omarorum* brain proliferation zones revealed by the double thymidine analog labeling technique. (A,B)** Projections of 25 confocal planes (sequentially acquired at one micron intervals) into a single plane of frontal sections at the level of the rombencephalic ventricle of fish treated with Protocol 1 **(A)** or 2 **(B)**. **(C,D)** Orthogonal projections in the *XZ* and *YZ* axes, crossing at the position indicated by the yellow arrows in **(A,B)**, respectively. As shown in **(A,C)**, a high proportion of proliferating cells was double-labeled after a short chase (24 h) indicating cell cycle reentry, and thus correspond to fast cycling cells (yellow arrow). Single labeled cells correspond to actively cycling cells at the moment of IdU (green arrow) and CldU (red arrow) exposure. Double labeled cells in **(B,D)** reentered the cell cycle after a long chase (1 month) and thus correspond to slow cycling cells which were scarce (yellow arrow). Note the pleiotropic nuclear staining, with varying labeling intensity and surface aspect (either smooth or grainy). Scale bars: **(A,B)** = 50 μm; **(C,D)** = 5 μm. Generalized green staining at the brain surface in **(B)** is due to non-specific fluorescence of remaining embedding gelatin/albumin mixture.

Two main types of nuclear labeling were found with both thymidine analogs, IdU and CldU, irrespective of the duration of the chase and the survival period after analog administration. Some nuclei showed homogeneous labeling while others presented granular staining. In both cases, the intensity of the labeling usually decreased with the duration of the post-thymidine analog administration survival. Nevertheless, few cells remained intensely stained in spite of a long post-thymidine analog survival. Both thymidine analogs stained similarly in double labeled nuclei, either granular or smooth. However, some double labeled nuclei showed homogeneous labeling of the first thymidine analog and granular labeling of the second (Figure 1). The shape of labeled nuclei also differed within and among proliferation zones, and as a function of post-thymidine analog survival. Nuclei located within the boundaries of the proliferation zones were generally round to ovoid at short survivals and ovoid to rod-like at long survivals, in frontal sections (Figure 1). Besides, nuclei were round to ovoid within some proliferation zones and elongated to rod-like within others, regardless of the survival exposure. The nuclei of most cells that appeared to have already migrated out of the proliferation zones were round and presented granular and weak staining, as reported in other teleosts.

Variations in the texture of nuclear staining (diffuse vs. grainy) might correspond to exposure to the thymidine analogs during early or late periods of the S-phase, as evidenced by confocal (Manders et al., 1992) and electron microscopy (Jaunin et al., 1998). In turn, variability in nuclear labeling intensity can be attributed to differences in the duration of thymidine analog exposure, the timing of analog exposure within the S-phase period (late S-phase exposure produces brighter labeling; Manders et al., 1992), and/or the duration of the cell cycle and the resultant amount of cell divisions between thymidine analog administration and animal fixation (labeling intensity decreases with subsequent cell divisions by replacement of exogenous thymidine analogs by endogenous thymidine; Zupanc et al., 2005; Alunni et al., 2010). Thus, proliferating cells with short cell cycles and hence high proliferative activity would show weaker label retaining than cells with long cell cycles or under a “wait” or quiescent state (Zupanc and Zupanc, 1992; Alunni et al., 2010). Variations in nuclear shape in frontal sections may be related to different states of the cells, as for example the rod-like nuclear shape of migrating newborn cells, and the round shape of already migrated nuclei (*D. rerio*: Zupanc et al., 2005; *O. latypes*: Alunni et al., 2010). Note that even though many nuclei had a circular shape in frontal sections, their three-dimensional shape was ellipsoidal or rod-like in some cases, and spherical in others, as evidenced by orthogonal projections (Figures 1C,D, respectively).

### Cellular composition of brain proliferation zones

Double immunohistochemical demonstration of IdU and CldU revealed three populations of proliferative cells, reflecting DNA replication in the presence of only one or both thymidine analogs, independently of the chase duration (Figure 1). Thus, single labeling demonstrated cells that were cycling at the moment of each analog administration (*actively cycling cells*), whereas double labeling evidenced cells that were cycling at the moment when each analog was administered, thus detecting cells that had reentered cell cycle (Nowakowski et al., 2002; Vega and Peterson, 2005; Kempermann, 2006; Llorens-Martín and Trejo, 2011). The use of two chase durations (short and long; Protocols 1 and 2, respectively) allowed us to further discriminate between proliferating cell types, based on the following criteria: (a) the labeling of the nucleus with both or only one thymidine analog (as indicator that the cell had or had not reenter the cell cycle, (b) the duration period of inter-thymidine analog administration, or post-thymidine analog survival, (c) the location of the nucleus (within or out of the proliferation zone), and (d) the intensity of the labeling (intense or weak). Considering these criteria, we identified at least four cell types: (1) Cells that reenter the cell cycle after a short chase, indicating short cell cycle duration and thus correspond to *fast cycling cells* (yellow arrows, Figure 1A). (2) Cells that reentered the cell cycle after a long chase, indicating long cell cycle duration and thus were considered *slow cycling cells* (yellow arrows, Figure 1B). (3) Cells that intensely retained the first thymidine analog after a long chase but did not incorporate the second thymidine analog, and remained within the boundaries of the proliferation zone (*long term intense thymidine analog label retaining cells*). These characteristics indicate that the cells did not divide or divided rarely after incorporating the first thymidine analog, and did not divide at the moment of the second thymidine analog administration. (4) Cells that faintly retained the first thymidine analog after a long chase (*long term weak thymidine analog label retaining cells)*, indicating that had cycled at the moment of the first thymidine analog administration, divided several times after that, but did not divide at the moment of the second thymidine analog administration. Cells of this type were usually found at diverse distances from the boundaries of the proliferation zones, evidencing that have already undergone a process of out-migration from the proliferation zones on other brain zones or subzones (arrowheads, Figure 1B). Nonetheless, some long term weak thymidine analog label retaining cells remain within the proliferation zones.

### Spatial distribution of adult brain proliferation zones

**Figure 2:**
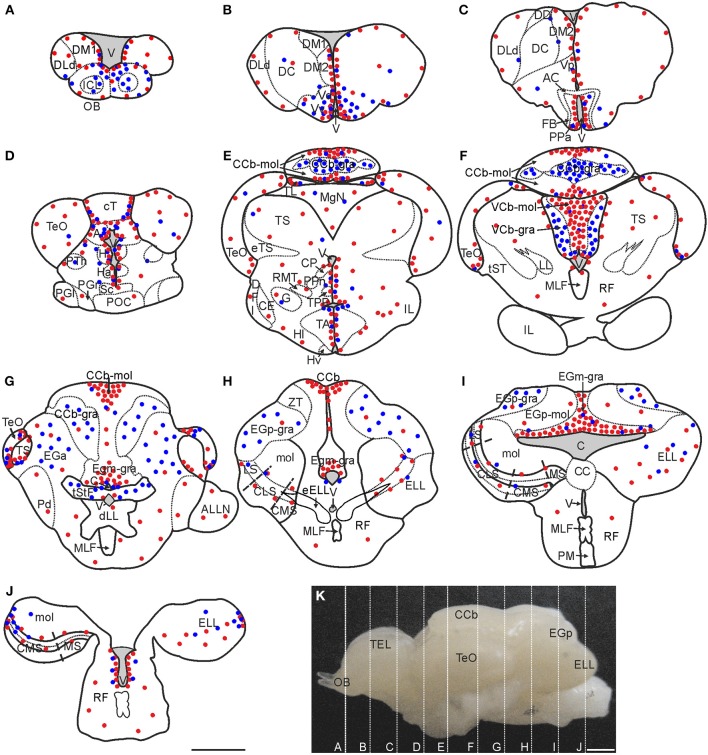
**Spatial distribution of proliferating cells in *Gymnotus omarorum* brain after a short (4 h; red dots) and long (30 days; blue dots) survivals after thymidine analog administration**. The distribution of proliferating cells, as revealed by thymidine analog labeling followed by a 4-h survival, is qualitatively represented by the red dots in the schematic diagrams of frontal sections **(A–J)** corresponding to the planes indicated in the lateral view of the brain **(K)**. Ventricular proliferation zones were found in the caudal olfactory bulb **(A)**, telencephalon **(A–C)**, diencephalon **(C–E)**, mesencephalon **(D–G)** and rombencephalon **(H–J)**. Extraventricular proliferation zones were found at the three divisions of the cerebellum (Cb): within the molecular layers of the corpus cerebelli (CCb-mol; **E–G**) and the valvula cerebelli (VCb-mol; **F**), and at the granular layer of the eminentia granularis pars medialis (EGm-gra; **G–I**). Another extraventricular proliferation zone was found at the lateral-caudal border of the electrosensory lateral line lobe (ELL; **J**). The distribution of long label retaining proliferating cells after a long post-thymidine analog survival is qualitatively represented by the location of the blue dots. Long range migration of long term weak thymidine label retaining cells was evident in the olfactory bulb (OB; **A**), subpallium (ventral telencephalon, dorsal subdivision -Vd-; ventral telencephalon, ventral subdivision -Vv-; **B**) and Cb [cerebellum, granular layer -CCb-gra- **(E,F)**; eminentia granularis pars anterior -EGa **(G)**, eminentia granularis pars posterior, granular layer -EGp-gra- **(H,I)**, and valvula cerebelli, granular layer -VCb-gra-**(F)**]. Intermediate to short range migration was found in the diencephalon **(C–E)**, optic tectum (TeO, **D–G**), torus semicircularis (TS, **G**), and ELL **(I,J)**. A, Pretectal nucleus; AC, Anterior commissure; ALLN, Anterior lateral line nerve; C, Cerebello-medullary cistern; CCb-mol, Cerebellum, molecular layer; CC, Crista cerebellaris; CCb, Corpus cerebelli; CE, Central nucleus of the inferior lobe; CLS, Centrolateral segment (ELL); CMS, Centromedial segment (ELL); CP, Central-posterior nucleus; cT, Tectal commissure; DC, Central division of dorsal forebrain; DD, Dorsal division of the dorsal forebrain; DFl, Nucleus diffusus lateralis of the inferior lobe; DLd, Dorsolateral telencephalon, dorsal subdivision; dLL, Decussation of lateral lemniscus; DM1, Dorsomedial telencephalon, subdivision 1; DM2, Dorsomedial telencephalon, subdivision 2; EGa, EGa-mol, Eminentia granularis pars anterior, molecular layer; EGp, Eminentia granularis pars posterior; EGp-mol, Eminentia granularis pars posterior, molecular layer; ELL, Electrosensory lateral line lobe; eELL, ELL efferents; eTS, Torus semicircularis efferents; FB, Forebrain bundle; G, Glomerular nucleus; H, Habenula; Ha, Hypothalamus anterioris; Hl, Hypothalamus lateralis; Hv, Hypothalamus ventralis; ICL, Internal cell layer; IL, Inferior lobe; LL, Lateral lemniscus; LS, Lateral segment (ELL); MgN, Magnocellular mesencephalic nucleus; MLF, Medial longitudinal fasciculus; mol, Molecular layer (ELL); MS, Medial segment (ELL); Pd, Nucleus praeminentialis dorsalis; PGl, Periglomerular nucleus, lateral subdivision; PGr, Periglomerular nucleus, rostral subdivision; PM, Pacemaker nucleus; POC, Postoptic commissure; PPa, Nucleus preopticus periventricularis, anterior subdivision; PPn, Prepacemaker nucleus; PTh, Nucleus prethalamicus; RF, Reticular formation; RMT, Rostral mesencephalic tegmental nucleus; Sc, Suprachiasmatic nucleus; TA, Nucleus tuberis anterior; TEL, Telencephalon; TL, Torus longitudinalis; TPP, Periventricular nucleus of the posterior tuberculum; tST, Subtectal tract; tStF, Tractus stratum fibrosum; V, Ventricle; Vp, Ventral telencephalon, posterior subdivision; ZT, Transitional zone. Scale bars: 1 mm.

To depict the spatial distribution of *G. omarorum* brain proliferation zones, we analyzed the localization of actively cycling cells after two short post thymidine analog administration survivals: 4 h after IdU (Protocol 1) and 24 h after CldU (Protocol 2). The distribution of proliferating cells in the brain of adult *G. omarorum* was uneven. Most labeled nuclei were found scattered in the midst of the gray matter throughout the whole brain; some nuclei were located within the white matter and others adjacent to the walls of blood vessels. Over this background, we identified several particular well-defined zones of brain tissue populated by densely packed proliferating cells (Figure 2) that, in agreement with descriptions of proliferation zones in *A. leptorhynchus* by Zupanc and Horschke (1995) conformed brain proliferation zones. As shown in Figures 1A,C, the location and extent of the proliferation zones did not appear to differ between postadministration survivals of 4 and 24 h, validating the use of both groups of results.

Brain proliferation zones were found at two main locations: either at the lining and subventricular zones of the ventricular-cisternal system (ventricular proliferation zones) or distant from the ventricular-cisternal system (extraventricular proliferation zones; Zupanc and Horschke, 1995; Ekström et al., 2001). Ventricular proliferation zones were found all along the rostral-caudal extension of the brain (Figure 2). The most striking extraventricular proliferation zones were found in all divisions of the cerebellum (Cb, Figures 2E–I). Another extraventricular proliferation zone was found at the most caudal portion of the lateral border of the ELL (Figure 2J). In other brain regions, such as the TeO and TS, actively cycling cells were found in zones that appear distant from the ventricular-cisternal system, but correspond to zones that were adjacent to the ventricles during development and thus are considered as remnants of ventricular proliferation zones (Figures 2F,G; Zupanc and Horschke, 1995; Hinsch and Zupanc, 2007; Zupanc, 2011).

#### Telencephalic proliferation zones

**Figure 3:**
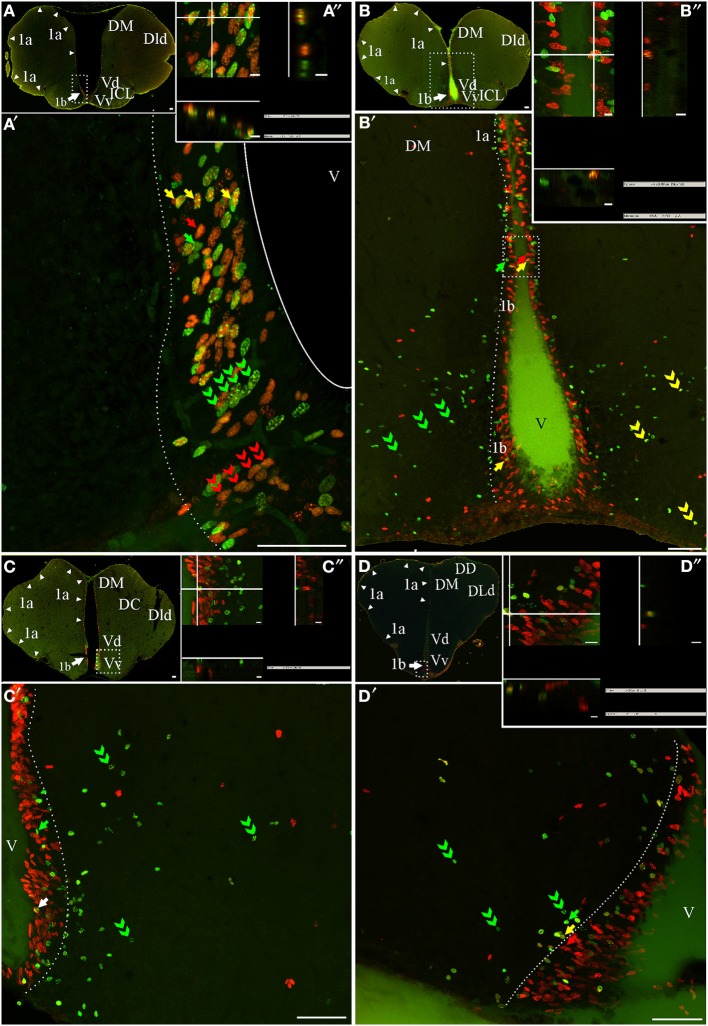
**Telencephalic proliferation zones: location and cellular composition**. Confocal images of frontal sections at rostral **(A–B)** intermediate **(C)** and caudal levels **(D)** of the telencephalic hemispheres and caudal levels of the olfactory bulb **(A,B)** of animals treated according to Protocol 1 **(A)** or 2 **(B–D)**. Images in **(A,A′–D,D′)** correspond to 30 sequentially acquired confocal planes (every 1 μm) projected to one plane. To corroborate the double labeling of nuclei, *XZ* and *YZ* orthogonal projections of the stacks were obtained at the sites indicated by the crossing lines **(A″–D″)**. The location and boundaries of three telencephalic proliferation zones, determined by the spatial distribution of actively cycling cells IdU^+^ (green arrows in **A′,A″**), CldU^+^ (red arrows in **A′–D′**) and **(A″–D″)**, were represented by arrowheads in **(A–D)** and dotted lines in (**A′–D′**): proliferation zone 1a at the free surface of the dorsal telencephalon; proliferation zone 1b at the ventral bulge of the telencephalic ventricle. Abundant double labeled nuclei were detected using Protocol 1 (fast cycling cells, yellow arrows in **A′,A″**), while few double labeled nuclei were found using Protocol 2 (slow cycling cells, yellow arrows in **B′,D′,B″,D″**). Note that some cells that appeared to be double labeled in the projected images (white arrow in (**C′**), in fact corresponded to neighboring cells at the same x-y position but at different focal planes and separately labeled with only one of the thymidine analogs **(C)**. Chains of elongated IdU+ or CldU+ nuclei are indicated by green and red double arrowheads, respectively. Long term weak analog label retaining cells are indicated by double green arrowheads in (**C′–D′**), indicating a long range radial migration. Scale bars: **(A–D,A′–D′)** = 50 μm; **(A″–D″)** = 5 μm. DC, Central division of dorsal forebrain; DD, Dorsal division of the dorsal forebrain; DM, Dorsomedial telencephalon; DLd, Dorsolateral telencephalon, dorsal subdivision; V, Ventricle; Vd, Ventral telencephalon, dorsal subdivision; Vp, Ventral telencephalon, posterior subdivision; Vv, Ventral telencephalon, ventral subdivision.

Similar to other vertebrates and teleosts, the telencephalon of *G. omarorum* comprises the paired olfactory bulbs (OB), the cerebral hemispheres (comprising a dorsolateral pallium and a ventromedial subpallium), and the caudal telencephalon impar (Nieuwenhuys, 2009). Regardless of the double thymidine labeling protocol used, only sparse proliferating cells were found near the free surface of the rostral and intermediate regions of the OB and did not appear to conform a clear proliferation zone (Figures 2A, 3A,B,B′). Conversely, in the intermediate portion of the OB, proliferating cells were grouped at its dorso-medial region, populating the lining of the most rostral portion of the telencephalic ventricle, forming a clear proliferation zone (Figure 2A). In frontal sections, labeled cells showed round to ovoid nuclei (data not shown).

The telencephalic hemispheres are solid and comprise two subdivisions, the subpallium or ventral telencephalon (formed by four regions: dorsal -Vd-, nother –Vn-, posterior –Vp-, and ventral –Vv; Figures 2B,C, 3A–D,B′–D′) and the pallium or dorsal telencephalon (formed by five regions: medial, dorsal, central, lateral (DL), and posterior; Figures 2A–C, 3A–D). DL is in turn sub-divided in dorsal, posterior, and ventral regions. Telencephalic labeled cells were contained in two ventricular proliferation zones, one facing all regions of the dorsal everted telencephalon (proliferation zone *1a*, Figures 2A–C, 3) and another facing all regions of the ventral telencephalon (proliferation zone *1b*, Figures 2B,C, 3). No clear boundaries were found either between the proliferation zone of the intermediate OB and proliferation zone *1b*, or between proliferation zone *1a* and proliferation zone *1b*.

The nuclei of proliferating cells adjacent to DD and DL were round to ovoid and sparsely distributed, while those adjacent to DM were round and densely packed (Figure 3B″). Most labeled nuclei of proliferation zone *1b* were elongated, densely packed, and frequently aligned, either perpendicularly or obliquely with respect to the ventricle surface. This arrangement resembles the appearance of migrating cells (green and red double arrowheads in Figures 3A′,B′). The extent of proliferation zone *1b* did not change with the duration of the post-thymidine survival from 4 to 24 h, as evidenced by the overlapped distribution of CldU^+^ and IdU^+^ nuclei using Protocol 1 (Figure 3A′). These indicate that a migration process is already occurring 24 h after CldU administration, but the duration was insufficient for derived cells to migrate out of the proliferation zone *1b* boundaries.

Both telencephalic ventricular proliferation zones showed a heterogeneous cellular composition. A short chase between analogs (Protocol 1, chase = 24 h) revealed double labeled nuclei of fast cycling cells (yellow arrows in Figures 3A′,A″) as well as actively cycling cells at the moment of each analog administration (CldU^+^ and IdU^+^: red and green arrows respectively in Figure 3A′). A long chase (Protocol 2) revealed rare double labeled nuclei of slow cycling cells (yellow arrows in Figures 3B′,D′) confirmed by orthogonal projections (Figures 3B″,D″), and few long term intense thymidine analog label retaining cells that remain within the proliferation zones (filled green arrows in Figures 3B′,C′).

#### Diencephalic proliferation zones

The diencephalon, flanked by the telencephalon and mesencephalon, includes the epithalamus, dorsal and ventral thalamus, and hypothalamus. According to Meek and Nieuwenhuys (1998) it also comprises the preoptic region and the synencephalic/pretectal region, despite their possible extra-diencephalic origin.

**Figure 4:**
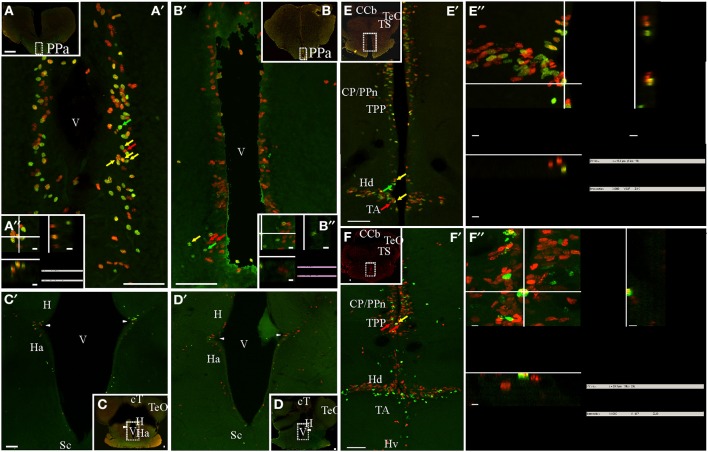
**Diencephalic proliferation zones: location and cellular composition**. Confocal images of frontal sections at rostral **(A,B)** intermediate **(C,D)**, and caudal levels **(E,F)** of the diencephalon of animals treated according to Protocol 1 **(A,C,E)** or 2 **(B,D,F)**. Areas indicated by the dotted rectangles are shown at higher resolution **(A′–F′)** as 30 sequentially acquired confocal planes (at 1 μm intervals) projected to one plane. To corroborate the double labeling of nuclei, *XZ* and *YZ* orthogonal projections of the stacks were obtained at the sites indicated by the crossing lines (**A″–D″,E′,F′**). Diencephalic proliferation zones were almost always restricted to the ventricular lining. In the most rostral diencephalic region, a noticeable proliferation zone lay underneath the anterior commissure, facing the anterior subdivision of the nucleus preopticus periventricularis (PPa, **A,B**). Further caudal, less populated proliferation zones were found at the lateral angles of the diencephalic ventricle that separates the habenula **(H)** from the hypothalamus anterioris (Ha) ventrally **(C,D)**. At the caudal-most region of the diencephalon **(E,E′,F,F′)**, there was an extended proliferation zone, covering almost continuously the lining of the hypothalamic ventricle and facing all hypothalamus divisions: dorsalis (Hd), lateralis (Hl), ventralis (Hv), and caudalis. Note the abundance of double labeled, fast cycling cells evidenced with Protocol 1 **(A′,A″,E′,E″)**, and the scarcity of double labeled, slow cycling cells, evidenced with Protocol 2 **(B′,B″,F′,F″)**. A clear migration process of long term weak labeling retaining cells was evident at the hypothalamic proliferation zone (double green arrow heads, **F′**). Scale bars: **(A–F,A′–F′)** = 50 μm; **(A″,B″,E″,F″)** = 5 μm.

The rostralmost diencephalic proliferation zone in *G. omarorum* was found at the level of the preoptic region (Figure 2C). This proliferation zone surrounded the preoptic recess of the diencephalic ventricle, extending from the anterior commissure to the optic chiasm and thus facing the anterior (PPa, Figures 2C, 4A,B) and posterior subdivisions of the nucleus preopticus periventricularis, and the suprachiasmatic nucleus (Sc; Figures 4C,D). Other diencephalic ventricular proliferation zones faced the pretectal nucleus A (A, Figure 2D) and the epithalamic habenula (Figures 2D, 4C′,D′), the dorsomedial thalamus and the diencephalic central-posterior nucleus (CP, Figures 2E, 4E′,F′) located medial to the prepacemaker nucleus (Figure 2E). Ventral to these regions, ventricular proliferation zones also faced, from rostral to caudal, the thalamus, the nucleus posterioris periventricularis and the periventricular nucleus of the posterior tuberculum (Figure 2E). An extended ventricular proliferation zone was found all along the walls of the hypothalamic ventricle, facing the hypothalamus anterioris (Figures 2D, 4C′,D′), dorsalis (Figures 4E′,F′) ventralis (Figures 2E, 4F′), lateralis (Figure 2E) and caudalis.

The appearance of labeled nuclei and spatial organization of proliferating cells varied between rostral (preoptic, Figures 4A,B), intermediate (epithalamic-thalamic, Figures 4C,D) and caudal (thalamic-hypothalamic, Figures 4E,F) regions of the diencephalic ventricular proliferation zones. Densely packed, rounded to ovoid nuclei populated the whole width of the ventricular lining along the entire rostral-caudal extension of the ventricular recess surrounded by the preoptic region (Figures 4A,B). The hypothalamic proliferation zones (Figures 4E,F) were characterized by densely packed proliferating cells that covered most of the extension of the ventricular lining. Conversely, at the intermediate part of the diencephalon, proliferating cells were less abundant and distributed in a patchy fashion.

Proliferating cells of the preoptic ventricular proliferation zone were round or ovoid and placed at varying distances from the preoptic recess' ventricular surface, a disposition that contributed to a pseudostratified appearance of the ventricular proliferation zone (Figures 4A,B). A short chase between thymidine analogs (24 h, Protocol 1) revealed the relative abundance of fast cycling cells (yellow arrows in Figures 4A′,A″) homogeneously distributed among actively cycling cells (red and green arrows in Figure 4A′). Conversely, a long chase (1 month, Protocol 2) evidenced scarce slow cycling cells (yellow arrows in Figures 4B′,B″).

Proliferating cells were rare at the intermediate portion of the diencephalic ventricle (Figures 4C,D), with the exception of the lining of the lateral recess of the diencephalic ventricle that separates the habenula from the hypothalamus anterioris (arrowheads in Figures 4C′,D′). At that location, there was an evident wedge-like thickening of the ventricular lining, populated by a small cluster of proliferating cells with elongated nuclei (Figures 4C′,D′). The cellular composition of this ventricular proliferation zone appeared different from other ventricular proliferation zones as neither Protocol 1 (Figure 4C′) nor Protocol 2 (Figure 4D′) identified cells reentering the cell cycle after a short or long chase.

An outstanding dorsal thalamic proliferation zone was evident at the ventricular lining facing the central posterior/prepacemaker nucleus complex (Figures 4E′,E″,F′,F″), and the periventricular nucleus of the posterior tuberculum (Figures 4E′,E″,F′,F″). This proliferation zone consisted of two to four sets of proliferating cells aligned parallel to the ventricular surface, including abundant fast cycling cells intermingled with actively proliferating cells (Protocol 1, either CldU or IdU labeled, Figures 4E′,E″) and few slow cycling cells (Figures 4F,F″). All proliferating cells showed round or elongated nuclei, whose main axes were perpendicular to the ventricular surface. At the same rostral-caudal brain level, a noteworthy proliferation zone populated both the dorsal and the ventral rims of the lateral recess of the hypothalamic ventricle (Figures 4E′,E″,F′) facing the hypothalamus dorsalis and the nucleus tuberis anterior, respectively. This proliferation zone also showed abundant fast cycling cells (yellow arrows in Figures 4E′,E″) intermingled with actively proliferating cells (red and green arrows (Figures 4E′,E″).

#### Mesencephalic proliferation zones

The mesencephalon comprises a dorsal midbrain roof, including the TeO and torus longitudinalis (TL), a ventrolateral TS, and a ventromedial tegmentum (Meek and Nieuwenhuys, 1998). All these brain regions presented particular proliferation zones in *G. omarorum*.

**Figure 5:**
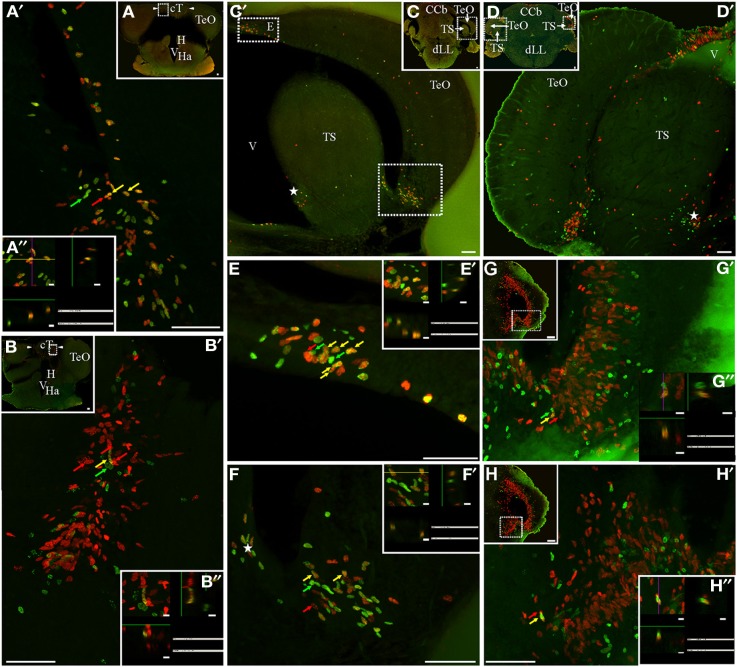
**Mesencephalic proliferation zones: location and cellular composition of tectum opticum (TeO) and torus semicircularis (TS) proliferation zones**. Confocal images of frontal sections at rostral **(A,B)** and caudal levels **(C,D,G,H)** levels of the mesencephalon of animals treated according to Protocol 1 **(A,C,E,F)** or 2 **(B,D,G,H)**. Images in **(A′–D′,E–H)** correspond to 30 sequentially acquired confocal planes (at 1 μm intervals) projected to one plane. To corroborate the double labeling of nuclei, *XZ* and *YZ* orthogonal projections of the stacks were obtained **(A″,B″,E″–H″)** at the sites indicated by the color coded arrows and the rectangles in the low power images. **(A,B)** The tectal commissure (cT) was flanked by two proliferation zones (arrowheads, **A,B**). Two groups of proliferating cells were evident in the dorsomedial and ventrolateral borders of the caudal part of the TeO **(C′,D′,E,F)** that converge into one at the caudal pole of the TeO **(G)**. A single proliferation zone was evident at the ventromedial region of the TS (asterisks in **C′,D′, F**). Note the relative abundance of fast cycling cells in cT **(A′,A″)** and TeO **(E,E′,F,F′)** and the scarcity of slow cycling cells in cT **(B′,B″)** and the caudal pole of TeO **(G′,G″)**. A shift in the distribution of IdU + and CldU+ actively cycling cells was found in cT proliferation zone **(B′)**. Scale bars: **(A–D,G,H, A′–H′)** = 50 μm; **(A″,B″,E″–H″)** = 5 μm.

The TeO is a cortical paired structure that covers most of the dorsal and lateral aspects of the mesencephalon. Both TeO converge at their rostral pole where they are interconnected by the tectal commissure (cT). The rostral portion of cT stands perpendicular to the main brain axis, parallel to the frontal plane, between the rostral poles of both TeO (Figures 5A,B); caudally, cT undergoes an almost 90° rotation so that it becomes flattened in the dorsal-ventral direction. A noticeable proliferation zone was evident at each border between the rostral portion of cT and the medial edge of the rostral pole of the TeO (Figures 5A,B). A short chase between thymidine analogs (Protocol 1, Figure 5A) revealed the relative abundance of fast cycling cells (yellow arrows in Figures 5A′,A″) intermingled with actively cycling cells at the moment of CldU (red arrows in Figure 5A′) and IdU (green arrows in Figure 5A′) administration. A long chase (Protocol 2) revealed scarce slow cycling cells (yellow arrow in Figures 5B,B′,B″). In each TeO we found a single horseshoe-shaped proliferation zone. Thus, in frontal sections the tectal proliferation zone looked as if it was formed by three independent proliferation zones, one at the narrow, dorso-medial border (Figures 2E–G, 5C–E), another at the blunt, ventro-lateral border (Figures 2E–G, 5C,D,F), and a third at the caudal pole of each TeO (Figure 5G). The tectal area diminished in the rostral-caudal direction, while the sectional area of the dorso-medial and ventro-lateral regions of the tectal proliferation zone enlarged. Thus, dorso-medial and ventro-lateral regions of tectal proliferation zone approach each other to coalesce at the caudal pole of TeO (Figure 5H). Proliferating cells were extended across the whole arc of the TeO caudal pole (Figure 5G). In all the extension of the tectal proliferation zone, proliferating cells occupied almost all TeO layers. As evidenced by Protocol 1, fast cycling cells were abundant (Figure 5C; yellow arrows in Figures 5E,F) and distributed among actively cycling cells (red and green arrows in Figures 5E,F,E′,F′). Conversely, slow cycling cells were very rare (yellow arrows in Figures 5G′,H′,G″,H″).

The TL, also a paired structure, lies at each side of the midline, underneath the horizontal portion of cT. TL is populated by densely packed granule cells that project to the ipsilateral superficial stratum marginale of the TeO; comprising a cerebellum-like circuit (Meek and Nieuwenhuys, 1998; Bell, 2002). A single-cell width proliferation zone covered the ventral ventricular surface of the TL (Figure 2E) all along its rostral-caudal extension, facing the obliterated mesencephalic ventricle (Figure 2E).

The TS is a prominent layered structure of the ventro-lateral tegmentum whose growth obliterates the mesencephalic ventricle in *Gymnotus*. We also found a proliferation zone at the medial-ventral and lateral-dorsal border of the TS (asterisks in Figures 5C′,D′,F), also converging at the caudal end of the TS (Figures 5H,H′). Note that, at the caudal pole of TeO and TS, both proliferation zones appear to be contiguous (Figures 5G,H). The cellular composition of TS proliferation zone was similar to that of TeO proliferation zone as it contained a high proportion of fast cycling cells and few slow cycling cells (yellow arrow in Figure 5H′).

#### Rombencephalic proliferation zones

The rombencephalon comprises a ventral, basal plate-derived motor zone, an intermediate visceral zone, and a dorsal alar plate-derived zone. The latter includes, rostrally, the cerebellum (Cb) and caudally, the somatosensory zone. The somatosensory zone consists of a general somatosensory trigeminal nucleus and a specialized somatosensory nucleus. In *G. omarorum*, like other weakly electric gymnotids, the rombencephalon is dominated by the specialized lateral line region, particularly the mechanosensory medial octavolateral nuclei and the ELL (Figures 2H–J). The Cb is formed by three main regions: a caudal or vestibule-lateral lobe, a central corpus (CCb, Figures 2E–G), and a rostral valvula cerebelli (VCb, Figure 2F), protruding into, and almost occluding, the midbrain ventricle. The vestibule-lateral lobe of Cb consists of a caudal lobe and two granular eminences, one anterior (Figure 2G) and another posterior, in turn subdivided into a medial granular eminence (Egm, Figures 2G,H) and a lateral-posterior granular eminence (Egp, Figures 2H,I) (Meek and Nieuwenhuys, 1998).

**Figure 6:**
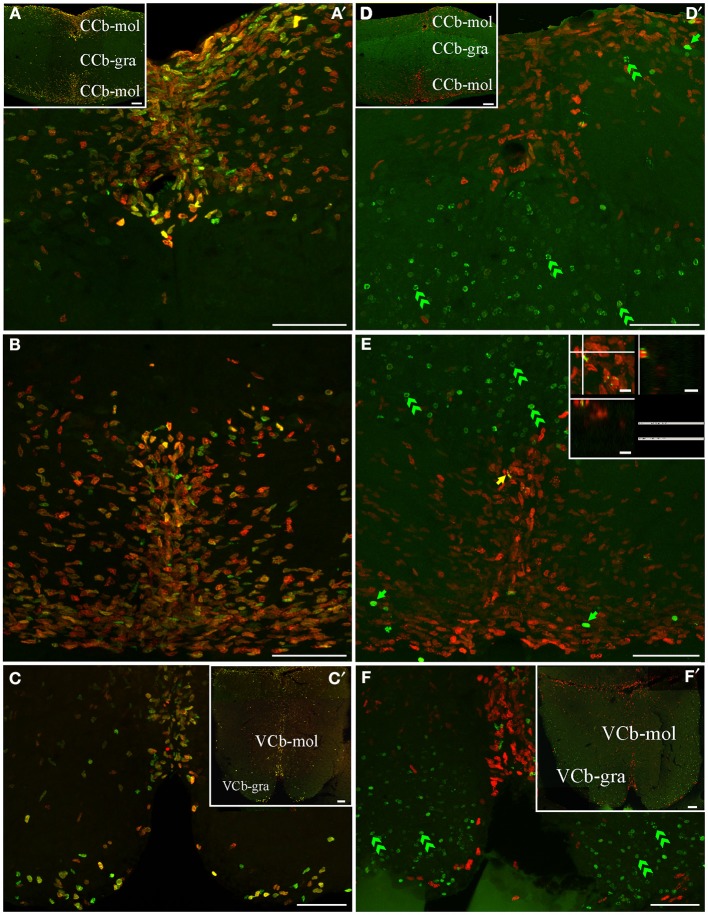
**Cerebellar (corpus cerebelli -CCb- and valvula cerebelli -VCb-) proliferation zones: location and cellular composition**. Confocal images of frontal sections at the level of the CCb **(A,B,D,E)** and VCb **(C,E)** of animals treated according to Protocol 1 **(A–C)** or 2 **(D–F)**. Images in **(A′,–C,D′–F)** correspond to 30 sequentially acquired confocal planes (at 1 μm intervals) projected to one plane; images **(A,D)** correspond to 15 sequentially acquired confocal planes (at 2 μm intervals) projected to one plane; images in **(C',F')** correspond to sequentially acquired images of a single plane. As shown by Protocol 1, densely packed fast cycling cells [double labeled cells in **(A–C)** were found at a band near the midline of the dorsal **(A)** and ventral **(B)** molecular layers of CCb (CCb-mol] and of the molecular layer of VCb **C**, VCb-mol). In animals treated according to Protocol 2, the proliferation zones of both cerebellar divisions showed few long term intense thymidine analog label retaining cells within the proliferation zone's boundaries; green arrows in **D–F**) and slow cycling cells (double labeled nuclei, yellow arrow in **E**), whereas abundant long term weak thymidine label retaining cells moved from CCb-mol and VCb-mol to the corresponding granular layers (double green arrowheads in **D–F**). Scale bars: **(A–D,A′–D′)** = 50 μm; **(A″–D″)** = 5 μm.

**Figure 7:**
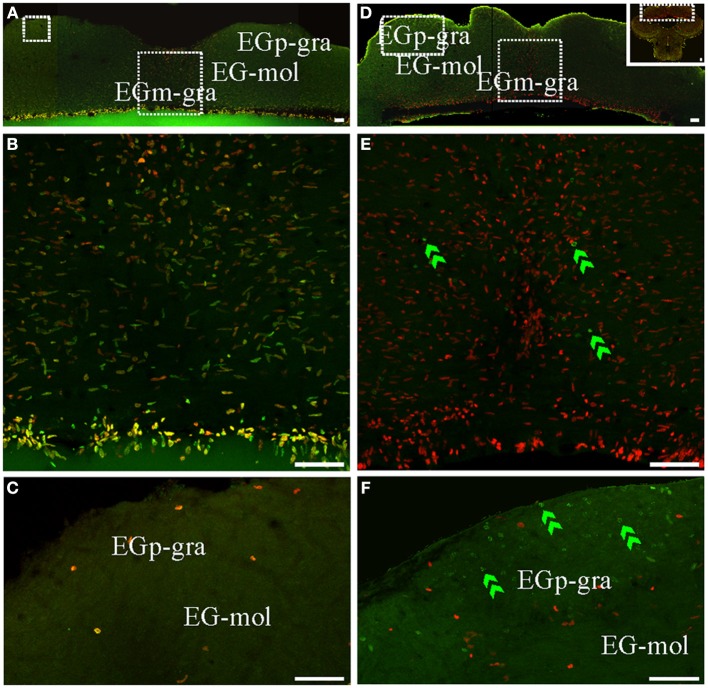
**Cerebellar (caudal lobe of Cb) proliferation zones: location and cellular composition**. Confocal images of frontal sections at the level of the caudal lobe of the Cb of animals treated according to Protocol 1 **(A–C)** or 2 **(D–F)**. **(A,B)** Show low power confocal images of the caudal lobe of the cerebellum that depict the location of caudal Cb proliferation zones. **(B–F)** Correspond to 30 sequentially acquired confocal planes (at 1 μm intervals) projected to one plane. Note that the granular layer of EGm (EGm-gra) was populated by actively cycling cells most of which corresponded to fast cycling cells (double labeled nuclei in **A,B**). Conversely, actively cycling cells were almost absent in both the molecular layer (EG-mol, **C**) and the granular layer of EGp (EGp-gra, **C**). Slow cycling cells were rare (not found in the shown images). Few long term weak thymidine label retaining cells were found throughout EGm-gra (double green arrowheads in **E**) but also migrated up to EGp-gra (double green arrowheads in **F**). Scale bars: 50 μm.

**Figure 8:**
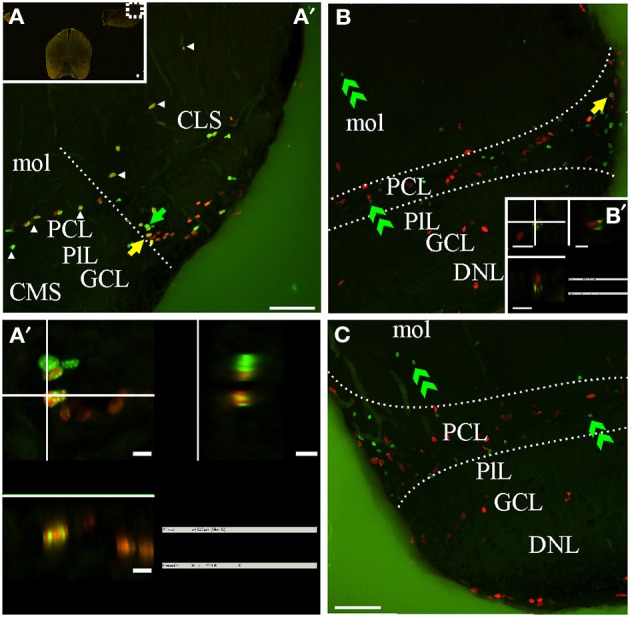
***G. omarorum*'s electrosensory lateral line lobe (ELL) proliferation zones**. Confocal images of frontal sections at caudal levels of the rombencephalon of animals treated according to Protocol 1 **(A,A′)** or 2 **(B,C)**. Images in **(A′,B,C)** correspond to 30 sequentially acquired confocal planes (at 1 μm intervals) projected to one plane. To corroborate the double labeling of nuclei, *XZ* and *YZ* orthogonal projections of the stacks were obtained at site indicated by the yellow arrow in (**A′–B**, inset in **B**). A group of proliferating cells was found at the lateral edge of the caudal pole of the ELL, near the border between the molecular (mol) and the principal cell layer (PCL) (delimited by dotted lines in **B,C**). Some proliferating cells were also aligned along most lateral-medial extension of the border between PCL and mol (arrowheads in **A**). Fast cycling cells were relatively abundant (yellow arrows in **A′,A″**), while slow cycling cells were scarce (note that the apparently double labeled nuclei in B in fact corresponds to a pair of nuclei, one IdU+ and the other CldU+ located at the same *X*-*Y* but different *Z* location as evidenced by the orthogonal projection in **B′**). At long chase, long term weak thymidine label retaining cells were found at the PCL **(B,C)**. Scale bars: **(A–C,A″)** = 50 μm; **(A″–B′)** = 5 μm.

An extended ventricular proliferation zone was localized along all the extension of the lining of the narrowed fourth ventricle and the cerebello-medullary cysternal system in adult *G. omarorum* (Figures 2G–J). In addition, extraventricular proliferation zones were found in the Cb (Figures 2E–I, 6, 7) and the ELL (Figures 2H–J, 8). The cerebellar extraventricular proliferation zone was the most extended and densely populated proliferation zone of *G. omarorum* brain. Each Cb division has its own proliferation zone. In the CCb, (Figures 2E–G, 6A,B,D,E) and the VCb (Figures 2F, 6C,F) the proliferation zones were circumscribed to the molecular layers. In both cases, the cellular density decreased in medial-lateral direction. Conversely, in the caudal lobe of the Cb, the proliferation zone was restricted to the granular layer of EGm, (EGm-gra, Figures 2G–I, 7). The EGp-gra, and the molecular layer of both EGm and EGp were almost devoid of proliferating cells. At the caudal pole of EGm, proliferating cells were aligned almost parallel to the brain surface. More rostrally, where the molecular layer widens and the brain expands in the dorsal-ventral direction, a band of proliferating cells could be observed extending along the midline from the dorsal edge of the EGm-gra up to the dorsal brain surface. Close to the brain surface, the proliferation zone expands in medial-lateral direction and thus acquires a triangular shape in frontal sections (Figure 2H). In all cerebellar divisions, the distribution of proliferating cells was homogeneous as evidenced both in frontal (Figures 6, 7B,E) and parasagittal sections (data not shown).

The ELL is a layered, cerebellum-like structure (Bell, 2002) that protrudes from the dorsal surface of the medulla, at each side and bellow the caudal lobe of Cb from which it is separated by a cistern (C, Figures 2I,J). The primary afferents that innervate cutaneous electroreceptors project somatotopically onto deeper layers of the ELL and contact secondary neurons; ELL principal cells project to the magnocellular mesencephalic nucleus or mesencephalic TS which projects back to the ELL, directly or via the Cb. ELL proliferating cells were sparse and usually aligned with the boundaries between ELL layers (Figures 2I,J, arrow in Figure 8A′) or grouped at the border between ELL segments (straight dotted line in Figure 8A′). At the caudal pole of the ELL, proliferating cells appear to be more abundant near the lateral edge of the boundary between the principal cell layer (PCL) and the molecular layer (mol). Thus, this proliferation zone usually appeared wedged-shaped in frontal sections (region between curved dotted lines in Figures 8B,C). Fast cycling cells were relatively abundant (yellow arrow in Figures 8A′,A″) whereas slow cycling cells were scarce or absent (Figure 8B′). At the same rostral-caudal level, a small number of proliferating cells were found in the ventral zone of the medulla, including the electromotor bulbar nucleus but did not appear to constitute a proliferation zone.

### Distribution of long term thymidine analog label retaining, newborn brain cells

As mentioned in section Cellular Composition of Brain Proliferation Zones, the use of a double labeling protocol with a long chase between thymidine analogs' administrations (Protocol 2; chase 30 day) allowed us to reveal the location of long term thymidine analog label retaining cells in the brain of adult *G. omarorum*. Among this cell type, few nuclei corresponded to long term intense thymidine analog label retaining cells that remained within the proliferation zones, while the majority presented the characteristics of long term weak thymidine analog label retaining cells that already traversed the proliferation zones limits. Thus, their distribution evidenced the likely migration of newborn cells.

Unlike short post-thymidine survivals, 30 days after thymidine analog administration several long term weak thymidine analog label retaining cells appeared within the OB, mainly at the internal cell layer (ICL) of both its intermediate (blue dots, Figure 2A) and rostral regions (not shown). Long term weak thymidine analog label retaining cells were frequent within the subpallium, lateral to the boundaries of the proliferation zone *1b*, and extending throughout adjacent Vd and Vv (blue dots, Figure 2B; double green arrowheads, Figures 3B′–D′). Contrarily, long term weak thymidine analog label retaining cells were rare throughout the pallium (DM, DD, DL, or DC) as mostly remained within the proliferation zone *1a* (Figures 2A–C) intermingled with actively cycling cells.

Within the rostral diencephalon, the long chase duration appeared to be sufficient for most long term weak thymidine analog retaining cells to migrate out the proliferation zone; only few cells remained within or near the ventral portion of the PPa after a chase of 30 day (blue dots, Figure 2C; green double arrow head, Figure 4B′). Conversely, in the caudal diencephalon, actively cycling cells were clearly segregated from long term weak thymidine analog retaining cells which appeared to have migrated from the dorsal to the ventral rim of the lateral recess of the hypothalamic ventricle (Figure 2E; green double arrow heads, Figure 4F′).

After 30 days of survival, long term weak thymidine analog retaining cells were rare throughout the mesencephalic TS and the TeO. At the rostral-most pole of the TeO, long term weak label retaining proliferating cells remained within cT proliferation zone, while others (Figure 2D; double green arrow heads, Figure 5B′) already dislodge from actively cycling cells (red arrows, Figure 5B′) at the medial-ventral end of cT proliferation zone). At the rostral region of tectal and toral proliferation zones, most long term weak label retaining proliferating cells were placed within or quite near the proliferation zones. No obvious migration of these cells was evidenced by Protocol 2 (Figures 2E,F; also compare the location of proliferating cells in Figures 5C,D, corresponding to the same rostral-caudal level of the TeO of animals treated according to Protocols 1 and 2, respectively). Thirty days after thymidine analog labeling only a short-range shift of long term weak thymidine analog retaining cells from deep to superficial layers of the caudal pole of the TeO was evidenced (double arrow heads, Figure 5G′).

Conversely, the distribution of long term weak thymidine analog retaining cells completely differed from that of actively proliferating cells after a chase of 30 day in all cerebellar divisions. Unlike actively cycling cells (Figures 6A–C), most long term weak thymidine analog retaining cells were found within the CCb-gra and VCb-gra, but only rarely in the corresponding molecular layers (Figures 2E,F; double arrow heads in Figures 6D–F). In the caudal Cb, a long range migration of long term weak thymidine analog retaining cells was also evident. Many cells of this type were abundantly and homogeneously distributed in the EGp-gra (double arrow heads, Figure 7F). Fewer long term weak thymidine analog retaining cells remained within the Egm-gra proliferation zone (Figures 2H,I; double arrow heads, Figure 7E).

The distribution of long term weak thymidine analog retaining cells in the caudal region of the ELL was almost coincident with that of actively cycling cells, occupying a band extending from the boundary between the mol and PCL, through the PCL up to the underlying plexiform layer, and from the ELL lateral border, up to the medial limit of this rombencephalic brain region. Some cells also migrated toward mol (double arrow heads, Figures 8B,C).

## Discussion

In this study we used thymidine analog labeling followed by a short survival, to identify and explore the distribution of actively cycling cells in the brain of adult *G. omarorum*, and test whether the proliferation zones described in late larvae (Iribarne and Castelló, 2014) persist during adulthood. To characterize the cellular composition the of adult brain proliferation zones, we evidenced main types of proliferating cells by means of the double thymidine analog labeling technique (Vega and Peterson, 2005). Finally, as a first attempt to study the fate of newborn cells, we examined the distribution of derived cells after a long survival period after thymidine analog labeling.

### Multiple proliferation zones persist in the brain of adult *G. omarorum*

Abundant proliferating cells were evident within all brain divisions of adult *G. omarorum*. The most remarkable proliferation zones were located adjacent to the telencephalic subpallium, the diencephalic PPa and hypothalamus, the mesencephalic TeO and TS, and all divisions of the rombencephalic Cb. Some proliferation zones were extensive (such as those of the Cb and hypothalamus) whereas others were discrete (as in TeO, TS, and ELL). The resemblance between the distribution of adult *G. omarorum* proliferating cells and larva's distribution (Iribarne and Castelló, 2014) indicates the persistence of a specific pattern of cell proliferation throughout postnatal life in the brain of *G*. *omarorum*. As occurs in other teleosts, this might subserve the continuous brain growth that accompanies indefinite body growth, along with the maintenance of the particular brain morphology (Zupanc and Horschke, 1995; Ekström et al., 2001; Candal et al., 2005b; Zupanc et al., 2005; Zupanc, 2006).

The relative position of the proliferation zones vary among brain regions. These differences might subserve diverse modes of growth due to differences in the spatial pattern of newborn cells' addition. The widespread and extended distribution of cerebellar proliferation zones, appear to enable its multidimensional growth (Zupanc, 2008). Proliferation zones restricted to the caudal regions of the ELL, TS, and TeO, may contribute to their continuous and concerted growth in rostral-caudal direction (Easter, 1983; Raymond and Easter, 1983; Lannoo et al., 1990). This may be relevant for the preservation of the functional organization of the electrosensory system since information processing involves the reciprocal connection between these somatotopically organized brain centers (Carr et al., 1981; Bastian, 1982; Heiligenberg and Dye, 1982; Krahe and Maler, 2014).

### Comparative aspects of distribution of adult brain proliferation zones

The spatial distribution of adult *G. omarorum* proliferation zones closely resembled those reported in other teleosts. In addition to the proliferation zones described in other weakly electric fish, we found a proliferation zone nearby the caudal-lateral border of the ELL of adult *G. omarorum*, a location still not reported in adults, but already described along the development of weakly electric gymnotids *Eigenmannia* (Lannoo et al., 1990) and *G. omarorum* (Iribarne and Castelló, 2014).

Proliferating cells were distributed all along the extension of the ventricular lining of the telencephalon in *G. omarorum*, with a prominent proliferation zone adjacent to the subpallium, similar to *G. aculeatus* (Ekström et al., 2001), *D. rerio* (Adolf et al., 2006; Grandel et al., 2006; Zupanc, 2011), *O. bonariensis* (Strobl-Mazzulla et al., 2010), A. burtoni (Maruska et al., 2012), *O. mossambicus* (Teles et al., 2012), *A. leptorhynchus* (in which the dorsal-lateral telencephalic division showed the largest amount of proliferating cells; Zupanc and Horschke, 1995), and *O. latipes* (Kuroyanagi et al., 2010). Very scarce proliferating cells were found in the OB of adult *G. omarorum*, most of which were located within the glomerular layer or at the dorsal-medial surface of the intermediate zone of the OB (corresponding to the rostralmost region of the telencephalic ventricle). This is consistent with the results reported in *D. rerio* (Byrd and Brunjes, 2001; Adolf et al., 2006; Grandel et al., 2006; Zupanc, 2011), *A. burtoni* (Maruska et al., 2012), and *O. mossambicus* (Teles et al., 2012). Conversely, Ekström et al. (2001) reported proliferating cells at the internal cell layer of *G. aculeatus*, and Fernández et al. (2011) found labeled cells in all OB laminae of *Austrolebias* (with the highest long term thymidine label retaining cells concentration in its medial and rostral part. Taking into account the scarce population of proliferating cells and its sparse distribution in most of *G. omarorum* OB, in our opinion, we did not find a clear proliferation zone, with the exception of the small cluster of proliferating cells located adjacent to the telencephalic ventricle. These are consistent with the findings of Grandel et al. (2006) in the OB of *D. rerio*.

The most conspicuous diencephalic proliferation zone of *G. omarorum* was found at the preoptic region, populating the lining of the preoptic recess of the diencephalic ventricle, facing the PPa. This is in agreement with the results reported in *A. leptorhynchus, O. latipes, N. Furzeri, A. burtoni*, and *O. mossambicus*, (Zupanc and Horschke, 1995; Kuroyanagi et al., 2010; Terzibasi et al., 2012; Maruska et al., 2012; Teles et al., 2012) and *D. rerio* (Zupanc et al., 2005; Zupanc, 2011). However, according to Adolf et al. (2006) and Grandel et al. (2006) this proliferation zone in *D. rerio* is restricted to the ventral portion of the diencephalic ventricle. Another outstanding diencephalic proliferation zone in *G. omarorum* was found at the ventricular lining of the hypothalamic ventricle, including all its recesses, similar to what was described in *G. aculeatus* (Ekström et al., 2001), *D. rerio* (Grandel et al., 2006), and A. burtoni (Maruska et al., 2012). The hypothalamic proliferation zone is less notorious in *A. leptorhynchus* (Zupanc and Horschke, 1995), *O. latipes* (Kuroyanagi et al., 2010), and *O. mossambicus* (Teles et al., 2012) and, and was not reported in *Austrolebias* (Fernández et al., 2011) and *B*. *gauderio* (Dunlap et al., 2011).

Within the mesencephalon of *G. omarorum*, the most outstanding proliferation zones were located in the TeO and the TS. Two poles of cell proliferation were found in the TeO. The most evident tectal proliferation zone was located along the dorsal-medial and ventral-lateral borders of the caudal third of the TeO, converging at its caudal pole. This pattern closely resembled those reported in *C. auratus* (Raymond and Easter, 1983), *O. latipes* (Nguyen et al., 1999; Kuroyanagi et al., 2010); *G. aculeatus* (Ekström et al., 2001), *O. mossambicus* (Teles et al., 2012), and *N. furzeri* (Terzibasi et al., 2012), but differed from those reported in *A. leptorhynchus* (Zupanc and Horschke, 1995), *Austrolebias* (Fernández et al., 2011), and *B. gauderio* (Dunlap et al., 2011). The comparison with *D. rerio* is hindered by the discrepancies in the descriptions of Zupanc (2011) and Zupanc et al. (2005) on one side, and Grandel et al. (2006) and Ito et al. (2010) on the other side. The pattern of proliferating cells' distribution in the TeO of *G. omarorum* appears closer to those described by Grandel et al. (2006) and Ito et al. (2010) in *D. rerio*. In the ventral border of the TL of *G. omarorum* a row of proliferating cells was also evident as occurs in *A. leptorhynchus* (Zupanc and Horschke, 1995), *Austrolebias* (Fernández et al., 2011) and *G. aculeatus* (Ekström et al., 2001). In *D. rerio*, Grandel et al. (2006) found proliferating cells at the ventricular surface of TL, while the graphical representation of TL proliferating cells in Zupanc et al. (2005) indicates a prevailing location at the medial and dorsal portion of this mesencephalic brain region. Another noteworthy proliferation zone in the mesencephalon of *G. omarorum* was located in the caudal pole of the TS, similar to *A. leptorhynchus* (zone X, Zupanc and Horschke, 1995) and *D. rerio* (Grandel et al., 2006), but different from *G. aculeatus* whose TS proliferation zone is more extended in rostral-caudal direction and covers most of the dorsal-lateral ventricular surface of TS (Ekström et al., 2001). Unlike these species, a diffuse distribution of proliferating cells was reported in the TS of *A. burtoni* (Maruska et al., 2012), *B. gauderio* (Dunlap et al., 2011), and *O. mossambicus* (Teles et al., 2012).

The most outstanding *G. omarorum* brain proliferation zone, both because of its extension and cellular density, was located in the rombencephalic Cb. Numerous densely packed proliferating cells were found in the three main regions of Cb, as occurs in other teleosts (Zupanc and Horschke, 1995; Zupanc et al., 1996; Ekström et al., 2001; Grandel et al., 2006; Ampatzis and Dermon, 2007; Kaslin et al., 2009; Delgado and Schmachtenberg, 2011; Fernández et al., 2011; Teles et al., 2012). The spatial distribution of proliferating cells of *G. omarorum* cerebellar proliferation zones very closely resembled those of *A. leptorhynchus* (Zupanc and Horschke, 1995; Zupanc et al., 1996) and *O. mossambicus* (Teles et al., 2012): proliferating cells populated the medial region of the dorsal and ventral portions of CCb-mol, the medial region of VCb-mol, and most of the extension of EGm-gra. These proliferation patterns differed from those of *O. latipes* (Kuroyanagi et al., 2010), *Austrobelias* (Fernández et al., 2011), and *C. aureatus* (Delgado and Schmachtenberg, 2011). In all cerebellar divisions, proliferating cells' density and proliferation zones' extension appeared to be greater in *G. omarorum* than any other species, including *A. leptorhynchus*. At the rostral end of CCb, proliferating cells are rare and restricted to a 150 μm wide strip in *A. leptorhynchus*, but very abundant and spread over a wider extension (about 300 μm) of the middle portion of CCb-mol in *G. omarorum*. In this species, a band of proliferating cells was even found at the rostralmost pole of the CCb, extending all along the dorsal-ventral extension of CCb-mol. Proliferating cells within the CCb and VCb proliferation zones of *G. omarorum* were homogeneously distributed as occurs in *A. leptorhynchus*, and not aligned along radial spokes, as occurs in *D. rerio* (Grandel et al., 2006; Kaslin et al., 2009) and *Austrobelias* (Fernández et al., 2011). These variations in cerebellar proliferation zones characteristics could be related to differences in the cerebellar cytoarchitecture among the compared teleost species.

Within the rombencephalon of *G. omarorum* we also found a discrete proliferation zone extending along the lateral border of the caudal pole of the ELL (adjacent to the lateral border of the principal and granular cell layers). This proliferation zone was not reported before in adult weakly electric gymnotids, even though Dunlap et al. (2011) found a lateral to medial gradient of proliferating cells in the ELL of adult *B. gauderio*. In adult *A. leptorhynchus*, ELL proliferating cells are evenly distributed along the medial lateral ELL extension (Zupanc and Horschke, 1995). In both species, proliferating cell density was also uneven in the dorsal-ventral l axis, showing higher values at a band extending from the granular cell layer to the pyramidal cell layers (*B. gauderio*, Dunlap et al., 2011) or the stratum fibrosum (*A. leptorhynchus*, Zupanc and Horschke, 1995). Conversely, two proliferation zones were identified in ELL during postnatal development of weakly electric fish: one medial, adjacent to the medial ampullary segment of the ELL (*Eigenmannia*: Lannoo et al., 1990), and another at the caudal-lateral border of the ELL *Eigenmannia*: Lannoo et al., 1990; *G. omarorum*: (Castelló and Iribarne, 2010; Iribarne and Castelló, 2014) by the lateral tuberous segments of the ELL.

In sum, the pattern of ventricular proliferation zones and extraventricular proliferation zones spatial distribution in *G. omarorum* resembled the distribution pattern reported in other teleosts, particularly in *D. rerio, G aculeatus, O mossambicus* and *A. leptorhynchus* (Zupanc and Zupanc, 1992; Zupanc and Horschke, 1995; Zupanc et al., 1996, 2005; Ekström et al., 2001; Adolf et al., 2006; Grandel et al., 2006; Teles et al., 2012). The striking similarities in the pattern of distribution of proliferating cells among evolutionary distant fish species provides evidence for a ground plan of brain proliferation patterning of euteleosts, contributing to the development and maintenance of the euteleostean brain bauplan, as pointed out by Grandel et al. (2006) and Kuroyanagi et al. (2010). We also found differences with non-electroreceptive-electrogenic fish, which can be attributed to differences in functional specialization (Zupanc and Horschke, 1995; Grandel et al., 2006). As mentioned before, our findings also differed from those of the weakly electric fish *A. leptorhynchus*, both in brain regions involved with electrosensory information processing and in brain regions with other functions.

### *G. omarorum* brain proliferation zones contain a heterogeneous cellular composition

The implementation of the sequential administration of two thymidine analogs along with their specific and simultaneous immunohistochemical demonstration, by means of already tested antibodies and visualization by confocal microscopy, allowed us to reveal the heterogeneous cellular composition of adult *G. omarorum* brain proliferation zones. According to Zupanc and Horschke (1995), once a thymidine analog is administrated, it is available for its incorporation into DNA for 2–4 h. In addition, Zupanc and Zupanc (1992) estimated the duration of ventricular proliferating cells cycle in 2 days, but also demonstrated that 10–30% of ventricular cells undergo mitosis within 12 h. Thus, the protocols we employed were appropriated to label both actively cycling cells at the moment of IdU and CldU administration, in addition to cells that progressed through successive S-phases, thus revealing their proliferative history (Vega and Peterson, 2005; Llorens-Martín and Trejo, 2011).

Taking into account previous descriptions of proliferating cells in diverse species (Alvarez-Buylla et al., 2001; Doetsch, 2003; Vega and Peterson, 2005; Adolf et al., 2006; Kuhn and Peterson, 2008; Ma et al., 2008; Tavazoie et al., 2008; Alunni et al., 2010; Llorens-Martín and Trejo, 2011) we propose a correlation between labeling patterns and types of proliferative/derived cells. As already shown, *G. omarorum* proliferation zones presented abundant cells that reenter the cell cycle after a short period and thus correspond to fast cycling cells. These features correspond to those of intermediate amplifier progenitors or transitory amplifying cells (Alunni et al., 2010). We also identified scarce cells that reenter the cell cycle after a long period. These long lasting label retaining, self-renewing progenitor cells can be attributed to stem cells, as shown in *D. rerio* telencephalon (chase: 19 days, Adolf et al., 2006), and *O. latipes* TeO (chase: 14 days; Alunni et al., 2010). The third cell type within the proliferation zones' boundaries were scarce and presented intense, homogeneous long term label retaining of only the first thymidine analog, indicating that cycled only at the moment of the first analog exposure. Thus, these cells might correspond either to cells that had entered a quiescent state or to stem cells that did not re-enter the cell cycle at the moment of the second analog administration (Zupanc and Zupanc, 1992; Alunni et al., 2010). A forth cell type faintly retained the first thymidine analog after a long post-thymidine survival; most of these cells were usually located outside the boundaries of the proliferation zones, and probably correspond to newborn neural cells that were undergoing migration from the proliferation zone to other brain regions or sub-regions.

We cannot assure the lineage of adult *G omarorum* newborn brain cells since we did not study the expression of neuronal or glial markers in conjunction with long term thymidine label retention. However, it is plausible that most of adult newborn cells in *G. omarorum's* brain correspond to the neuronal lineage, and a minor proportion to the glial lineage, as occurs in other teleosts, even though the proportion of newborn neurons and glial cells varied among species, brain regions and sub-regions, and duration of the survival after thymidine analog administration (Zupanc et al., 1996, 2005; Adolf et al., 2006; Hinsch and Zupanc, 2007; Fernández et al., 2011; Teles et al., 2012; Terzibasi et al., 2012). In fact, scarce neurogenesis -evidenced by co-localization of thymidine analogs and the neuronal marker Hu- was found as early as 1 day (*Austrolebia*s telencephalon, mesencephalon and rombencephalon; Fernández et al., 2011), 3 days (*D. rerio* dorsal telencephalon; Adolf et al., 2006), and 7 days (*N. furzeri* telencephalon, TeO and Cb; Terzibasi et al., 2012). Fifteen days after BrdU administration, the proportions of newborn neurons in OB and telencephalon of *D. rerio* reaches 40 and 75%, respectively (Adolf et al., 2006). Almost 50% of the total amount of proliferating cells differentiate into neurons at a survival period of 270–744 days (Zupanc et al., 2005; Hinsch and Zupanc, 2007), even though a great variation among brain regions exists (hypothalamus: 7%; OB: 9%; TeO: 12; telencephalon: 42;%; Hinsch and Zupanc, 2007). Similarly, Teles et al. (2012) found a high and variable proportion of adult born brain cells expressing Hu in *O. mossambicus* 100 d after BrdU administration (dorsal telencephalon: 41–56%; OB glomerular and granular layers: 19 and 58%; VCb-mol and VCb-gra: 44 and 80%).

On the other hand, the proportion of newborn glia, as evidenced by co-localization of thymidine analogs and the glial markers GFAP or S-100, is low in the adult brain of teleosts after long lasting post-thymidine survival (*D. rerio*; 1%; Zupanc et al., 2005; *O. mossambicus*: dorsal telencephalon: 14–24%; OB glomerular and granular layers: 41 and 14%; VCb-mol and VCb-gra: 19–10%; Teles et al., 2012).

Further studies in progress, combining double thymidine analog labeling technique with immunohistochemistry for neuronal and/or glial epitopes, or with tract tracing neuronal labeling, will be necessary to show the fate of newborn cells and reveal the neurogenic potential of adult *G. omarorum* brain.

### Migration of derived cells

We looked for evidence of migration by newborn cells derived from brain proliferation zones, by combining a long postIdU (30 days) and a short postCldU (24 h) survival (Protocol 2). This allowed us to simultaneously reveal the boundaries of the proliferation zones (short term label retaining CldU positive cells) and the position of derived newborn cells (long term label retaining IdU positive nuclei) relative to the location of both, the proliferation zones and the surrounding brain regions in adult *G. omarorum*.

We found differences in the migratory behavior of derived cells. In some brain regions the distribution of IdU and CldU labeled cells almost overlapped, indicating a very slow pace of migration of derived cells, if any at all. This was the case of pallial proliferation zone *1a*. In other brain regions, as for example the caudal pole of the TeO and TS, we found a slight shift in the spatial distribution of CldU and IdU labeled cells, indicating a short range or slow pace of migrating cells. The same protocol also revealed a clear segregation of the spatial distribution of CldU and IdU labeled cells in the subpallial proliferation zone *1b* and the three divisions of Cb providing evidence for middle to long range and faster pace of migration of newborn cells. In fact, we found long term weak analog label retaining cells at distances as much as 200 μm lateral from the subpallial proliferation zone *1b*, indicating a radial migration of subpallial proliferation zone-derived cells into Vd, Vv and the caudalmost portion of the ICL as shown in *D. rerio* (Adolf et al., 2006; Grandel et al., 2006). Besides, we also found newborn cells within the intermediate and rostral region of the OB, suggesting a tangential migration from proliferation zone 1b into the OB. Preliminary results indicate that newborn cells migrated into the OB and differentiate into neurons at longer survivals after CldU administration (Lasserre, 2014), further supporting the conserved characteristics of the rostral migratory stream among anamniotes (Adolf et al., 2006; Kishimoto et al., 2011; Teles et al., 2012) and amniotes including humans (Lim et al., 2008; Wang et al., 2011).

In all Cb divisions, derived cells also showed long range and/or relatively fast migration: derived cells almost completely shifted their position between cerebellar layers (from CCb-mol to CCb-gra, VCb-mol to VCb-gra, or even from EGm-gra, through EGp-mol, up to EGp-gra) after a post-thymidine analog survival of 1 month. Nuclei of already migrated cells were usually round and presented weak and granular staining, unlike those of proliferating cells within cerebellar proliferation zones, indicating that derived cells had undergone several cell divisions and thus had partly substituted the exogenous thymidine analog by endogenous thymidine before or during migration. All these features argue in favor of differentiation of cerebellar newborn cells into granular cells as is also indicated by preliminary results of simultaneous demonstration of long term label retaining and the expression of neuronal markers or “*in vivo”* tract tracing in *G. omarorum* (Olivera-Pasilio, 2014) as shown in other teleosts (Zupanc et al., 1996, 2005; Kaslin et al., 2009; Delgado and Schmachtenberg, 2011; Teles et al., 2012).

The comparative analysis of proliferation zones' distribution and fate of newborn neural cells is considered an important input for the understanding of the functional role and of the mechanisms that control adult neurogenesis along evolution (Lindsey and Tropepe, 2006; Sullivan et al., 2007; Kaslin et al., 2008; Grandel and Brand, 2013). Several proliferation zones here described in the brain of adult *G. omarorum* deserve particular attention because of their potential as source of newborn neural cells, many of which may differentiate into neurons that finally will be incorporated into pre-existing neural circuits. Such is the case of the ventricular proliferation zone adjacent to the subpallium which might be the source of newborn neurons that migrate tangentially up to the OB -and thus constitute the anamniote counterpart of the amniote rostral migratory stream- (Adolf et al., 2006; Grandel et al., 2006) or radially to the adjacent Vd and Vv, the teleostean homologous of the amniotes striatum and septum (Ganz et al., 2012; Harvey-Girard et al., 2013). Interestingly, although in mammal striatum does not occur neurogenesis “*in vivo*,” and the SVZ gives rise to neuroblasts that migrate to the OB, striatal ischemic lesions induce the generation of neuroblasts from the SVZ that migrate in chains into the striatum (Yamashita et al., 2006). The comparative relevance of migration and/or potential neurogenesis in other brain regions (ELL, TS, TeO, and Cb) emerge from their involvement in sensory information processing, motor control and/or sensory-motor integration. The potential incorporation of newborn neurons at different relay stations of the neural circuits involved in electrosensory information processing may have functional consequences on the computational power of the network and thus, in the functional specialization of *G. omarorum* brain.

In conclusion, in this work we provide further evidences that adult cell proliferation is a conserved feature of euteleosts. In fact, the spatial distribution of adult *G. omarorum* brain proliferation zones is similar to closely related as well as phylogenetically distant species within the euteleostean radiation. We also demonstrated the heterogeneous cellular composition of all analyzed *G. omarorum* adult brain proliferation zones, and the ubiquitous presence of putative stem cells. Our results also revealed the profuse generation of newborn cells, whose pace of migration varied among proliferation zones. Further studies in progress, combining double thymidine analog labeling technique with immunohistochemistry to evidence the expression of neuronal markers or with tract tracing, will reveal the cellular identity of newborn cells. This will allow us to further envisage the functional role of adult cell proliferation and neurogenesis.

### Conflict of interest statement

The authors declare that the research was conducted in the absence of any commercial or financial relationships that could be construed as a potential conflict of interest.
