# Supplementary figures and images for: Spatial distribution and cellular composition of adult brain proliferative zones in the teleost, Gymnotus omarorum
Source: Front Neuroanat. 2014 Sep 8;8:88. doi: 10.3389/fnana.2014.00088 (PMC4157608; doi:10.3389/fnana.2014.00088)

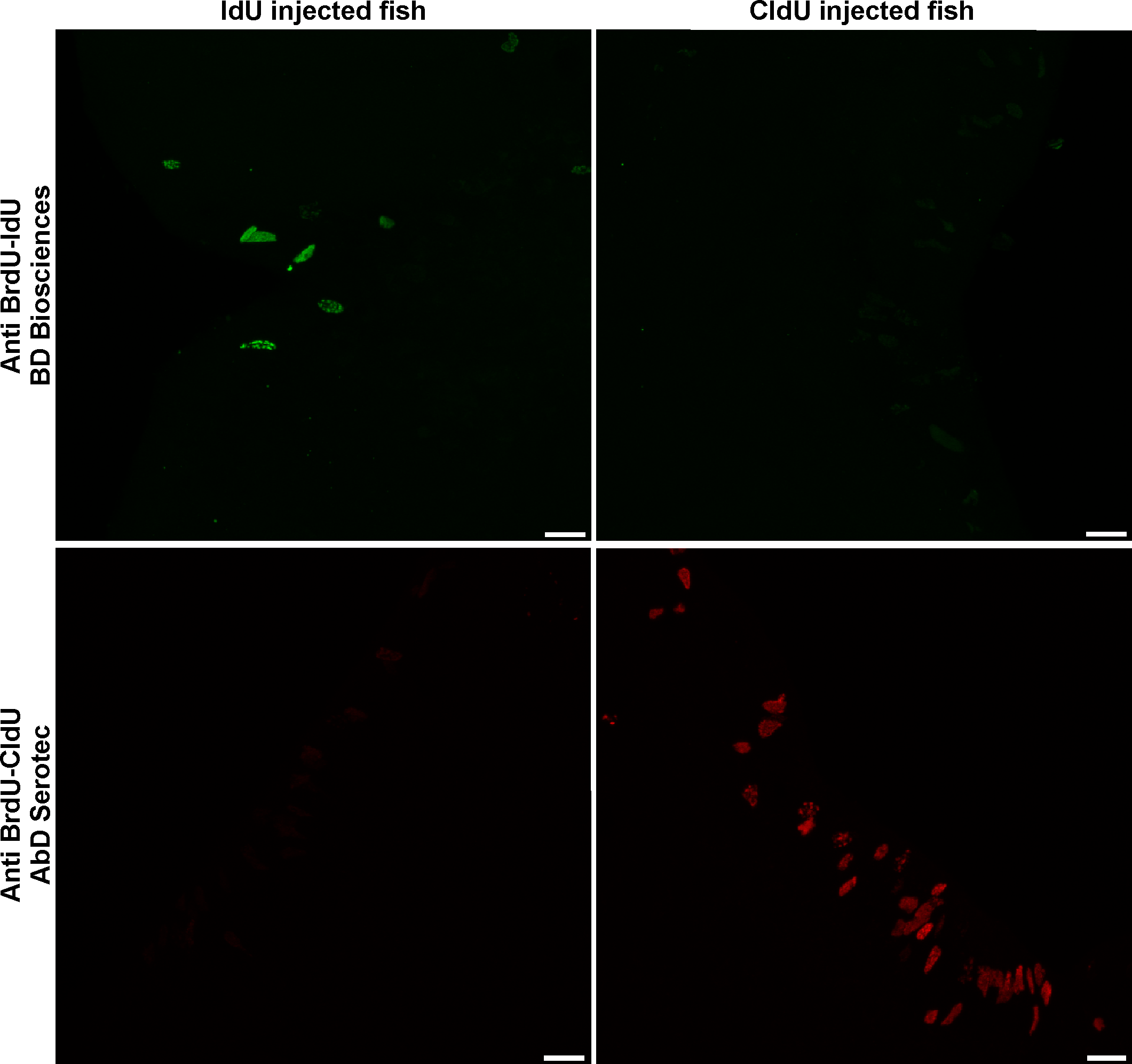

Supplement: Supplementary file 1 [file Image1.TIF]
